# First comprehensive analysis of lysine succinylation in paper mulberry (*Broussonetia papyrifera*)

**DOI:** 10.1186/s12864-021-07567-5

**Published:** 2021-04-10

**Authors:** Yibo Dong, Ping Li, Ping Li, Chao Chen

**Affiliations:** 1 College of Animal Science, Guizhou university, Guiyang, 550025 Guizhou China; 2 Department of Plant Protection, Institute of Crop Protection, College of Agriculture, Guizhou University, Guiyang, 550025 Guizhou China; 3 Institute of Grassland Research, Sichuan Academy of Grassland Science, Chengdu, 610000 Sichuan China

**Keywords:** Paper mulberry, Lysine succinylation, Posttranslational modification, Photosynthesis

## Abstract

**Background:**

Lysine succinylation is a naturally occurring post-translational modification (PTM) that is ubiquitous in organisms. Lysine succinylation plays important roles in regulating protein structure and function as well as cellular metabolism. Global lysine succinylation at the proteomic level has been identified in a variety of species; however, limited information on lysine succinylation in plant species, especially paper mulberry, is available. Paper mulberry is not only an important plant in traditional Chinese medicine, but it is also a tree species with significant economic value. Paper mulberry is found in the temperate and tropical zones of China. The present study analyzed the effects of lysine succinylation on the growth, development, and physiology of paper mulberry.

**Results:**

A total of 2097 lysine succinylation sites were identified in 935 proteins associated with the citric acid cycle (TCA cycle), glyoxylic acid and dicarboxylic acid metabolism, ribosomes and oxidative phosphorylation; these pathways play a role in carbon fixation in photosynthetic organisms and may be regulated by lysine succinylation. The modified proteins were distributed in multiple subcellular compartments and were involved in a wide variety of biological processes, such as photosynthesis and the Calvin-Benson cycle.

**Conclusion:**

Lysine-succinylated proteins may play key regulatory roles in metabolism, primarily in photosynthesis and oxidative phosphorylation, as well as in many other cellular processes. In addition to the large number of succinylated proteins associated with photosynthesis and oxidative phosphorylation, some proteins associated with the TCA cycle are succinylated. Our study can serve as a reference for further proteomics studies of the downstream effects of succinylation on the physiology and biochemistry of paper mulberry.

**Supplementary Information:**

The online version contains supplementary material available at 10.1186/s12864-021-07567-5.

## Background

The posttranslational modification of proteins refers to the chemical modification of proteins after translation; these modifications can regulate the activity, localization and folding of proteins and their interactions with other biological macromolecules [1]. An increasing number of studies have shown that posttranslational modifications (PTMs) are major determinants of the structure of chromatin and play key roles in the regulation of functional gene expression profiles to enhance the diversity of protein species and functions of organisms [2, 3]. Lysine is one of the amino acids in proteins that is the most frequent site of posttranslational modifications [4], such as acetylation [5], ubiquitination [6], butyrylation [7], malonylation [8] and succinylation.

Succinylation is a newly identified protein posttranslational modification (PTM) of lysine residues [9]. The succinyl group is covalently bound to the lysine residue via the succinyl donor through an enzymatic or nonenzymatic reaction [10]. Lysine succinylation was first observed in histone proteins and was found to play a role in regulating gene transcription [11]. Lysine succinylation has also been observed in proteins in the cytoplasm [12], nucleus and mitochondria [13], revealing that lysine succinylation regulates various important biological processes, including the cell cycle, growth and signal transduction pathways [14]. Recent studies have identified global lysine succinylation sites at the proteomic level in microorganisms, animals, humans and plants [15–18], demonstrating that succinylation is ubiquitous in diverse organisms. Subsequent studies verified histone lysine succinylation in prokaryotes [19] and eukaryotic cells [20], and more comprehensive lysine succinylome studies in humans, yeast, mice and bacteria have confirmed that Ksuc is evolutionarily conserved and ubiquitous [21, 22]. Hundreds of succinylation sites and proteins have been identified in a variety of microorganisms. Various bacteria [23] and fungi, such as *Escherichia coli*, *Mycobacterium tuberculosis* and *Toxoplasma gondii*, have been shown to undergo succinylation [24, 25]. A systematic analysis of mammalian succinylation sites in mouse cells [26] indicated that protein succinylation sites may affect enzymes involved in mitochondrial metabolism. Succinylation has also been studied in the human tissue/cell proteome [27]. Accurate identification of succinylation sites can facilitate our understanding of the molecular mechanism and potential roles of lysine succinylation [28]. Lysine succinylation has also been identified in plants. A total of 710 Ksu sites in 346 proteins with diverse biological functions and subcellular localizations were identified in rice (*Or. sativa, cultivar Nipponbare*) samples [4]. Moreover, studies have shown that other plants also have complete succinylation systems. For example, a total 605 lysine succinylation sites in 262 proteins were observed in *Brachypodium distachyon* L. leaves [29] and 3530 lysine succinylation sites in 2132 proteins were detected in white tea (*Camellia sinensis (L.) O. Kuntze*) [30]. A total of 347 lysine succinylation sites in 202 proteins were identified in tomato (*Solanum lycopersicum*) by high-resolution mass spectrometry [31], 416 lysine succinylation sites in 277 proteins were identified in wheat (*T. aestivum L.*) [32], and modified proteins were involved in a variety of biological processes. However, the understanding of succinylation in plants remains limited.

Paper mulberry (*Broussonetia papyrifera*) is an efficient traditional Chinese medicine and a tree species important for urban and rural afforestation. Paper mulberry is fast growing, strongly adaptable, and widely distributed. It reproduces easily and has a short rotation period. The paper mulberry leaves can be used as protein feed, the bark is a high-quality raw material for papermaking, and the roots, stems, leaves, fruits, and seeds can be used as medicines with substantial economic value [33]. Previous research has reconstructed the metabolic pathways of paper mulberry and analyzed the differentially expressed genes in the leaves and roots using transcriptomic data [34]. In the analysis, biosynthesis; photosynthesis; and metabolism were the top three KEGG enrichment pathways. Proteomics studies have also shown that energy metabolism is essential for the paper mulberry’s resistance to external stress. Previous reports have shown that lysine succinylation is involved in important processes such as plant biosynthesis and the regulation of metabolic pathways [35]. Therefore, we hypothesized that lysine succinylation could play an important role in the development and metabolism of paper mulberry. However, studies of lysine succinylation in paper mulberry have not been conducted at the proteomic level. Lysine succinylation may play an important role in paper mulberry development and metabolism. To test this hypothesis, we performed a proteomic study.

## Methods

### Plant material and growth conditions

**Fig. 1:**
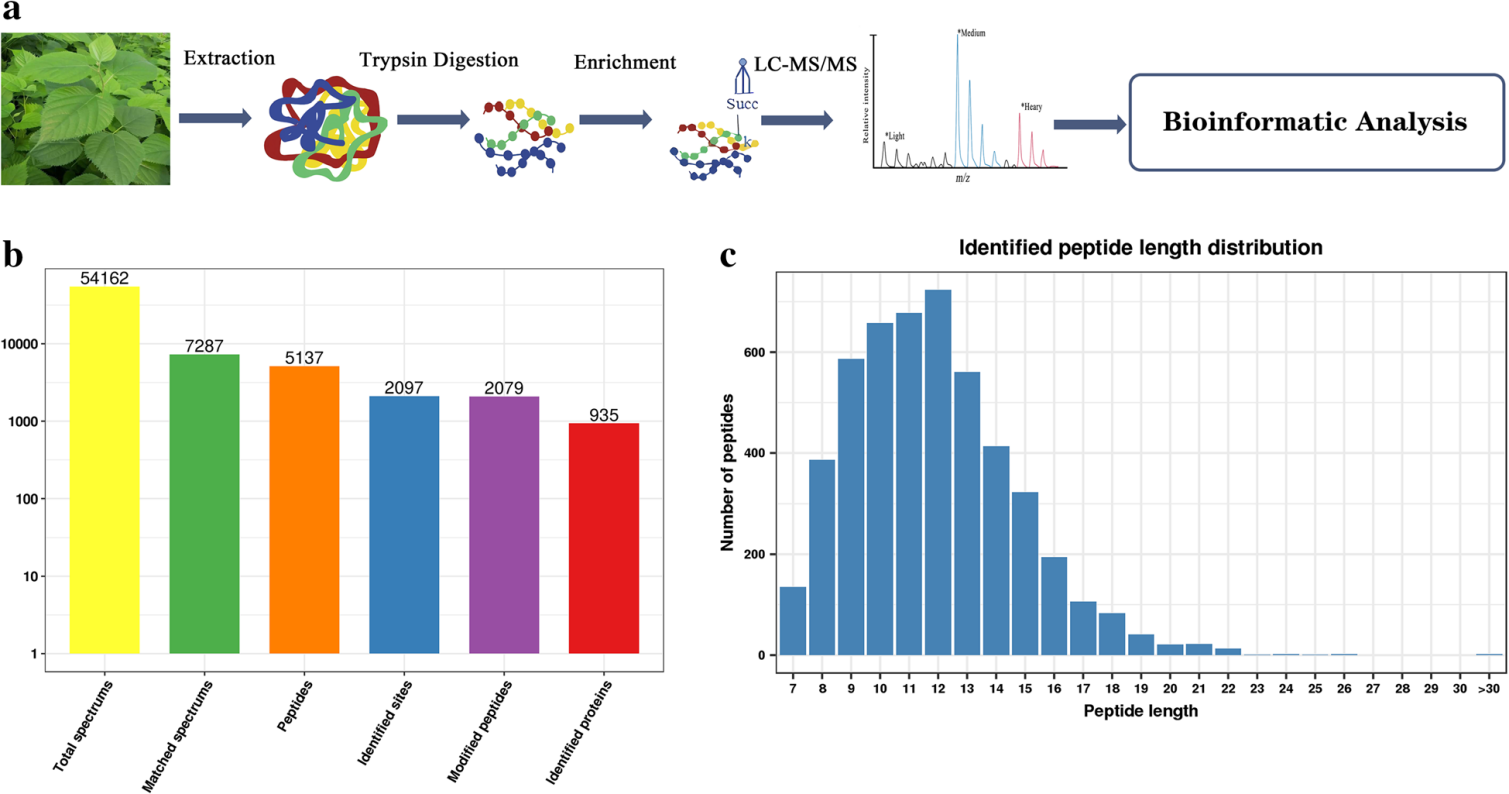
Identification of global succinylation sites and proteins in paper mulberry leaves. **a** General workflow of succinylation analysis. **b** Length distribution of the peptides. **c** Basic statistical table of MS results

Paper mulberry plants (Zhong Ke 1) were introduced from the planting base of Hainan Zhongbroussonetia Agriculture and Animal Husbandry Ecological Science and Technology Development Co., Ltd. to Zhenfeng County, Guizhou Province. Paper mulberry seedlings were transplanted by the authors in pots in a greenhouse of the College of Animal Science, Guizhou University, Guizhou Province, China (26°25^′^39.62^″^N, 106°40^′^5.81^″^E, 1090 m above sea level). The soil type was limestone with a pH of 7.72. The paper mulberry seedlings were grown in a greenhouse at 26/18 °C (day/night) and a photoperiod of 16/8 h (light/dark). Three biological replicates of 15 g of leaves were harvested from 7-week-old seedlings for protein extraction. The samples were immediately frozen in liquid nitrogen and stored at − 80 °C. The overall technological process is shown in Fig. 1a.

### Protein extraction and trypsin digestion

Leaf samples were initially ground with liquid nitrogen and washed with Trichloroacetic acid (TCA). Then, the powder was transferred to a 5-mL centrifuge tube and sonicated three times on ice using a high-intensity ultrasonic processor (Scientz, Ningbo, China) in lysis buffer containing 1% Triton X-100, 10 mM dithiothreitol, 1% protease inhibitor cocktail (Calbiochem, Darmstadt, Germany) 50 μM PR-619, 3 μM TSA (Sigma, California, USA), 50 mM NAM (Sigma, California, USA) and 2 mM EDTA. An equal volume of Tris-saturated phenol (pH 8.0) was added; then, the mixture was vortexed for 5 min. After centrifugation (at 5000 g at 4 °C for 10 min), the upper phenol phase was transferred to a new centrifuge tube. The Proteins were precipitated by adding four volumes of ammonium sulfate-saturated methanol, followed by incubation at − 20 °C for at least 6 h. After centrifugation at 4 °C for 10 min, the supernatant was discarded. The remaining precipitate was washed once with ice-cold methanol followed by three washes with ice-cold acetone [36]. One mL of 0.1 M ammonium ester dissolved in 80% methanol was added to the above centrifuge tube and swirled until thoroughly mixed. After centrifugation (4 °C, 5000 g, 10 min), the solution was washed with 1 mL of 80% TCA and shaken with a vortex until the sediment at the bottom of the tube was completely dispersed. After centrifugation (4 °C, 16000 g, 3 min), the solution was blown in a fume hood for 10 min to remove the acetone. The sample (0.1 g) was added to 0.8 mL 1:1 phenol (pH 8.0, Sigma, California, USA), mixed completely and incubated for 5 min at 4 °C. After centrifugation, the supernatant of phenol (0.2–0.4 mL) was transferred to a new 2 mL centrifuge tube, and 0.1 M ammonium acetate methanol solution was added, and stored overnight at − 20 °C. The protein was redissolved in 8 M urea, and the protein concentration was determined using a BCA kit (Beyotime, Shanghai, China) according to the manufacturer’s instructions.

For digestion, the protein solution was reduced with 5 mM dithiothreitol for 30 min at 56 °C and alkylated with 11 mM iodoacetamide for 15 min at room temperature in the dark. The protein sample was then diluted to a urea concentration of less than 2 M by adding 100 mM TEAB. Finally, trypsin was added at a trypsin to protein mass ratio of 1:50 for the first digestion overnight and 1:100 trypsin for the second 4-h digestion.

After trypsin digestion, the peptides were desalted by passage through a Strata X C18 SPE column (Phenomenex, Tianjin, China) and vacuum-dried. The peptides were initially separated into 60 fractions using a gradient from 8 to 32% acetonitrile (pH 9.0) over 60 min. Then, the peptides were combined into 4 fractions and dried by vacuum centrifugation.

### Pan-antibody-based PTM enrichment

To enrich the succinylated peptides, we dissolved the tryptic peptides in NETN buffer (pH 8.0) consisting of 1 mM EDTA, 100 mM NaCl, 0.5% NP-40 and 50 mM Tris-HCl, followed by incubation with previously washed antibody beads (PTM-402, PTM Bio, Hangzhou, China) overnight at 4 °C with mild shaking. The beads were then rinsed four times with NETN buffer and twice with H_2_O. The bound peptides were eluted from the beads with 0.1% trifluoroacetic acid. Finally, the eluted fractions were combined and vacuum-dried. Prior to LC-MS/MS, C18 ZipTips (Millipore, Massachusetts, USA) were utilized to desalt the peptides in accordance with specific protocols.

### LC-MS/MS analysis

The modified peptides were dissolved in 0.1% formic acid (solvent A) and directly loaded onto a homemade reversed-phase analytical column (15 cm length, 75 μm i.d.). The gradient included an increase from 6 to 22% solvent B (0.1% formic acid in 98% acetonitrile) over 20 min, from 22 to 30% over 8 min and to 80% over 2 min; then, the mobile phase was maintained at 80% for 2 min at a constant flow rate of 250 nL/min using a NanoElute 1000 UPLC system [37].

The peptides were subjected to an NSI source followed by tandem mass spectrometry (MS/MS) using TimsTOF Pro (Bruker, Karlsruhe, Germany) coupled online to the UPLC system. The electrospray voltage was 1.4 kV. The m/z scan range was 100 to 1700 for the full scan [38]. A data-dependent procedure alternated between one MS scan followed by 10 MS/MS scans with 5.0-s dynamic exclusion.

### Database search

We used the MaxQuant search engine (v.1.5.2.8) to process the obtained MS/MS data. In addition, we searched the tandem mass spectra from the paper mulberry protein database focusing on the reverse decoy database. We deemed trypsin/P a lyase that allowed for as many as 4 missing cleavages. The mass tolerance for precursor ions was set to 20 ppm in the initial search and 5 ppm in the main search, and the mass tolerance for fragment ions was set to 0.02 Da. In addition, we defined carbamidomethyl on Cys and Met oxidation as fixed and variable modifications, respectively. Moreover, we adjusted at < 1% and set the lowest score of modified peptides at > 40.

### Bioinformatics analysis

The motif-x (http://motif-x.med.harvard.edu/) algorithm was used to analyze the model of sequences composed of amino acids in specific positions of modify-21-mers (10 amino acids upstream and downstream of the modification site; however, phosphorylation used modify-13-mers with 6 amino acids upstream and downstream of the modification site) in all protein sequences [39]. Gene Ontology (GO) annotation proteome was obtained from the UniProt-GOA database (http://www.ebi.ac.uk/GOA/). The proteins were classified by GO annotation into three categories: biological process, cellular compartment and molecular function. For each category, a two-tailed Fisher’s exact test was used to test the enrichment of the identified modified proteins versus all the proteins in the species database. GO with a corrected *p*-value < 0.05 was considered significant [18]. The Kyoto Encyclopedia of Genes and Genomes (KEGG) database was used to identify enriched pathways by a two-tailed Fisher’s exact test to analyze the enrichment of the identified modified proteins versus all the proteins in the species database [40, 41]. Interproscan, software for searching the interpro database (http://www.ebi.ac.uk/interpro/) based on the sequence ratio, was used for protein domain annotation. WOLF PSORT was used to predict the subcellular localization of the succinylated proteins in paper mulberry. All the differentially expressed modified protein database accessions or sequences were searched against the STRING database [42] version 10.5 to assess protein-protein interactions. Only interactions between the proteins included in the searched dataset were selected, thus excluding external candidates. STRING calculates a metric called the “confidence score” to define interaction confidence; all the interactions that had a confidence score > 0.7 (high confidence) were retrieved. The interaction network based on STRING was visualized by the R package “networkD3”.

## Results and discussion

### Motif analysis of lysine succinylation sites in paper mulberry

PTMs play an important role in regulating cell biology because PTMs can change the physical or chemical properties, activity, localization or stability of a protein. Extensive studies have aimed to identify PTMs and determine their biological functions [43]. Paper mulberry is a multifunctional tree species widely used in papermaking, feed, medicine and other industries. In this study, a qualitative analysis of lysine succinylation was performed to assess its physiological and biological effects in paper mulberry. A total of 5137 peptides were detected, including 2079 succinylated peptides (Additional file 1: Table S1). A total of 2097 succinylation sites located on 935 proteins were identified (Fig. 1b). As shown in Fig. 1c, most of the peptides contained 7–20 amino acids, in agreement with the general features of trypsin-based enzymatic hydrolysis and HCD fragmentation.

Only the leaves of the tree were selected for analysis in this study. Considering that succinylation is tissue-specific [11], additional tissues and organs should be considered. The levels of protein succinylation differ significantly across various species (Table 1), which may explain the discrepancies between the results of this study and previous studies.

**Table 1:** Lysine succinylome identified in paper mulberry and other plants

Lysine succinylation	Numbers of identified proteins	Numbers of identified sites	Reference
Paper mulberry	935	2097	this study
Taxus (*Taxus×media*)	193	325	[39]
Rice (*Oryza sativa*)	347	854	[18]
Tea	86	142	[30]

### Properties of peptide succinylation sites

**Fig. 2:**
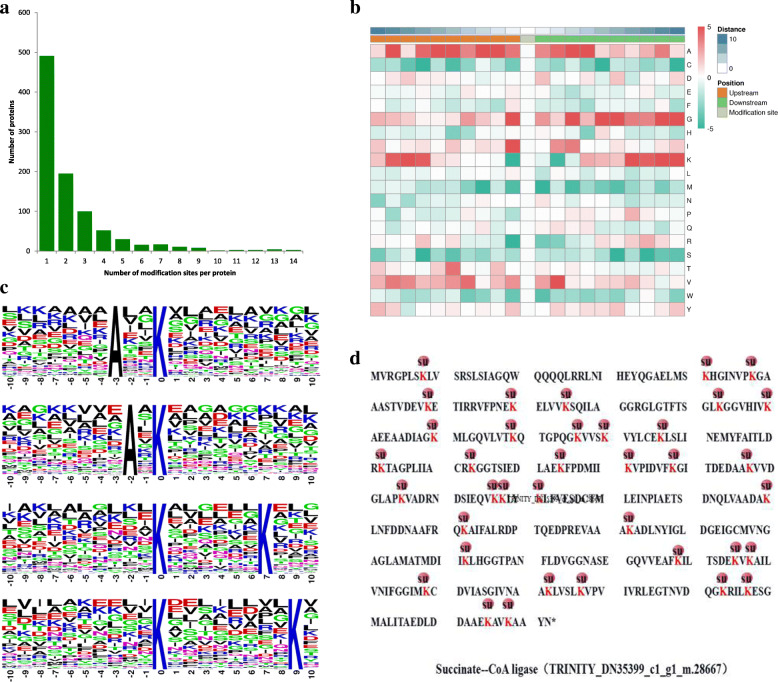
Motif analysis of the detected succinylation sites. **a** Length distribution of the peptides. **b**. Motif enrichment heatmap of the upstream and downstream amino acids of all identified modification sites. **c**. Enriched acetylation motif logos. The size of each letter represents the frequency of the amino acid residue at that position

To evaluate the properties of lysine succinylation sites in paper mulberry, the sequence motifs in the identified proteins were analyzed using Motif-x software. All 2097 acetylation sites were included in the following analysis. The number of modification sites varied from 1 to 14 per protein (Fig. 2a). Four conserved sequences with amino acids from − 10 to + 10 surrounding the succinylated lysine were extracted (Fig. 2b). The protein motifs were used for statistical analysis of the sequences of amino acids around the succinylation sites in the samples, and trends in the amino acid sequences around the succinylation sites were calculated. The motifs included A*Ksuc, A**Ksuc, Ksuc******K and Ksuc********K (Ksuc represents lysine succinylation, and * indicates a random amino acid residue) (Fig. 2c). The identification of two sequences containing alanine (A) suggested that A may be a common amino acid downstream of the succinylation site, while the identification of two sequences containing lysine (K) indicated that K may be a common amino acid upstream of the succinylation site.

Based on the motif characteristics of lysine succinylation in mulberry, the frequencies of A and K are slightly higher in each position around a succinylated lysine (− 10 to + 10): A (alanine) at position − 3, A (alanine) at position − 2, K (lysine) at position + 7, and K (lysine) at position + 8. The heatmap results also showed that the frequencies at − 3, − 2, + 7 and + 8 were significantly higher. Therefore, proteins with specific amino acid residues are more likely to be modified by succinylation. Notably, two succinylation motifs, Ksuc******K and K********Ksuc, were also detected in tea [44] and tomato [31], confirming that lysine succinylation is a highly conserved posttranslational modification in various species. The PTM modification site located in a representative protein, succinate-CoA ligase (TRINITY_DN35399_c1_g1_m.28667), was also succinylated (Fig. 2d). The level of cellular Ksu fluctuates with the in vivo level of succinate-CoA [15]. In this study, succinate-CoA synthesis catalysis in vivo is a biomarker for central metabolic recovery.

### Function annotation and subcellular location analysis

**Fig. 3:**
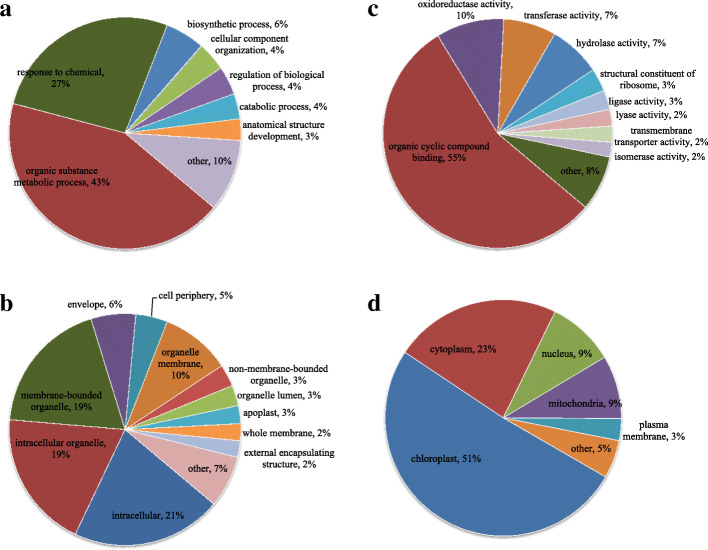
Functional distribution and subcellular localization of lysine-succinylated proteins in paper mulberry. **a**. Classification of succinylated proteins based on biological processes. **b**. Classification based on cellular component. **c**. Classification based on molecular function. d Subcellular localization of succinylated proteins

To determine the potential function of succinylation in mulberry, a structural analysis of all the identified proteins was performed. The GO annotations were classified into three categories, including biological process, cellular component and molecular function (Fig. 3; Additional file 2: Table S2); these classifications were used to explain the biological functions of the proteins from different perspectives. Then, the distribution of the proteins with identified modification sites among the GO secondary annotations was analyzed.

Classification analysis of biological process indicated that most of the succinylated proteins are involved in metabolic processes (43%), response to stimulus and stress (27%), biosynthetic processes (6%), and cellular component organization (4%) (Fig. 3a). The results in the cellular component category showed that most succinylated proteins are distributed in the categories of cells (21%), organelles (19%), membrane-bound organelles (19%), cell membranes (10%) and other (7%) (Fig. 3b). The molecular function analysis included proteins related to binding and oxidoreductase activities as the major succinylated proteins in the paper mulberry plants, accounting for 55 and 10% of all identified proteins in this category, respectively (Fig. 3c). The subcellular localization of the succinylated proteins was also investigated. As shown in Fig. 3d, most of the succinylated proteins in paper mulberry are distributed in the chloroplast (51%), cytoplasm (23%), mitochondria (9%) and nucleus (6.9%). The results of the GO functional classification and subcellular location analysis suggested that succinylated proteins are located in diverse cellular compartments in mulberry and participate in various biological processes.

The functional classification and subcellular location distribution of succinylated proteins were compared in three plants (paper mulberry, patchouli [45] and tea [30]). The cellular components of the three species are characterized by considerable enrichment in categories related to the cell, membrane, macromolecular complex and organelle membranes. Catalytic activity (various enzymatic reactions) and binding activities were the top two terms at the molecular function level. In the three plants, succinylated proteins are mainly distributed in the chloroplast, cytoplasm and nucleus.

### Functional enrichment analysis of succinylated proteins

**Fig. 4:**
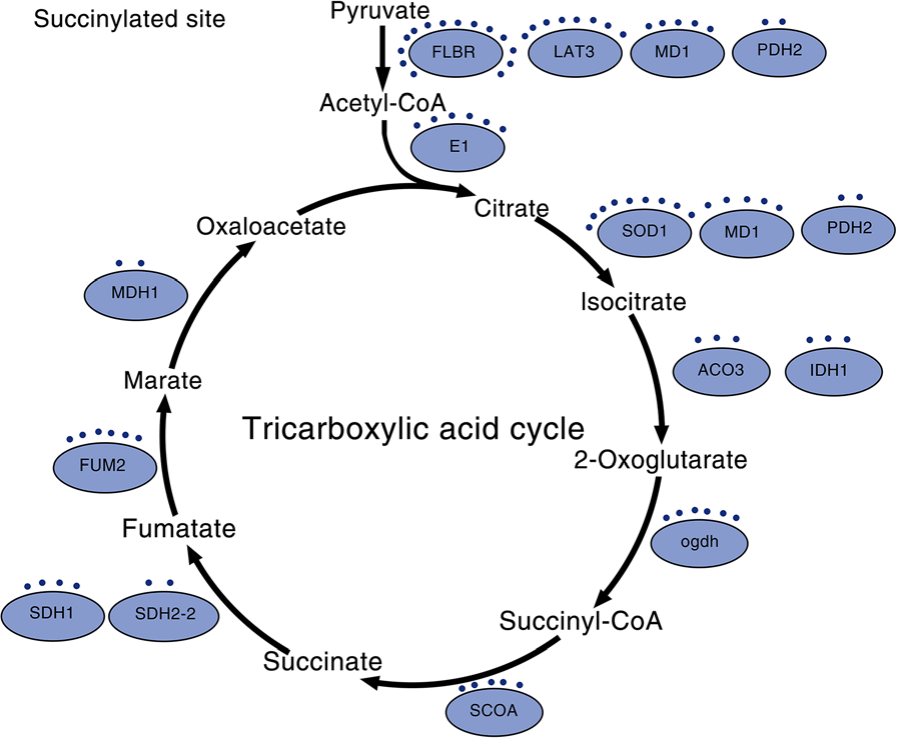
Succinylated enzymes involved in the TCA cycle. The enzymes include dihydrolipoyllysine-residue acetyltransferase component 1 of the pyruvate dehydrogenase complex (LAT2 and LTA3), malate dehydrogenase (MD1), pyruvate dehydrogenase E1 component subunit alpha-1 (E1), leghemoglobin reductase (FLBR), pyruvate dehydrogenase E1 component subunit (PHD2), aconitate hydratase 3 (ACO3), 3-isopropylmalate dehydrogenase (IDH1), violaxanthin de-epoxidase (VDE1), 2-oxoglutarate dehydrogenase (ogdh), succinate-CoA ligase [ADP-forming] subunit (SCOA), succinate dehydrogenase [ubiquinone] flavoprotein subunit (SDH1) and succinate dehydrogenase [ubiquinone] iron-sulfur subunit 2 (SDH2–2)

Functional enrichment analyses based on GO, KEGG pathways and protein domains were performed to determine the characteristics of succinylated proteins in paper mulberry in detail. As shown in Fig. 4a, the results of GO enrichment showed that most succinylated proteins are involved in energy production, material metabolism and biosynthesis processes. Tricarboxylic acid metabolic process, monocarboxylic acid metabolic process, citrate metabolic process and ribonucleoside monophosphate metabolic process are the top four biological processes, suggesting that lysine succinylation influences glucose metabolism and respiration. Several ribonucleoside monophosphate metabolic processes, purine nucleoside monophosphate metabolic process and pyruvate metabolic process terms were also highly enriched. In agreement with these observations, the top four GO terms (plastid stroma, chloroplast part, plastid part and plastid) are related to chloroplasts, suggesting that chloroplast metabolism-related enzymes may be targets and substrates of lysine succinylation. Additionally, the photosynthetic membrane and thylakoid membrane are directly related to photosynthesis and were significantly enriched. In the molecular function category, cobalt ion binding, copper ion binding, ion binding and oxidoreductase activity were the top four enriched terms.

KEGG pathway analysis identified twenty significantly enriched pathways (Fig. 4b and Additional file 3: Table S3), including the citric acid cycle (TCA cycle), carbon fixation in photosynthetic organisms, glyoxylate and dicarboxylate metabolism, photosynthesis, fatty acid degradation, oxidative phosphorylation and peroxisomes, suggesting a regulatory role of succinylation in the metabolism of diverse materials. The TCA cycle and oxidative phosphorylation play important roles in organisms because these pathways are the primary sources of energy. The TCA cycle also leads to primary metabolism and secondary metabolic pathways of various substrates and intermediates [46]. The remaining significantly enriched pathways were mainly related to the metabolism or biosynthesis of various secondary metabolites, such as glyoxylate and dicarboxylate metabolism, and multiple amino acid (pyruvate, valine, leucine, isoleucine, alpha-linolenic acid, alanine, aspartate and glutamate) degradation. Notably, these pathways are related to amino acid metabolism. Protein pathway enrichment analysis indicated that the top two significantly enriched domains were thioredoxin and pyridine nucleotide-disulfide oxidoreductase proteins. The ATP synthase alpha/beta family and nucleotide-binding domain were also enriched (Fig. 4c).

### Lysine succinylation in the chloroplast and cytoplasm of paper mulberry

Photosynthesis is one of the most essential metabolic processes in paper mulberry. Chloroplasts are the specific energy conversion organelles of plant cells [47, 48] and convert light energy into chemical energy to support plant life. Previous studies of lysine succinylation in wheat [49], patchouli plant leaves [45], *Brachypodium distachyon* L. [29] and tomato [31] identified various succinylation sites and proteins related to photosynthesis and demonstrated potential functional regulation of photosynthesis-related proteins by succinylation. Thus, protein succinylation may be a conserved form of regulation of photosynthesis in various plant species.

**Fig. 5:**
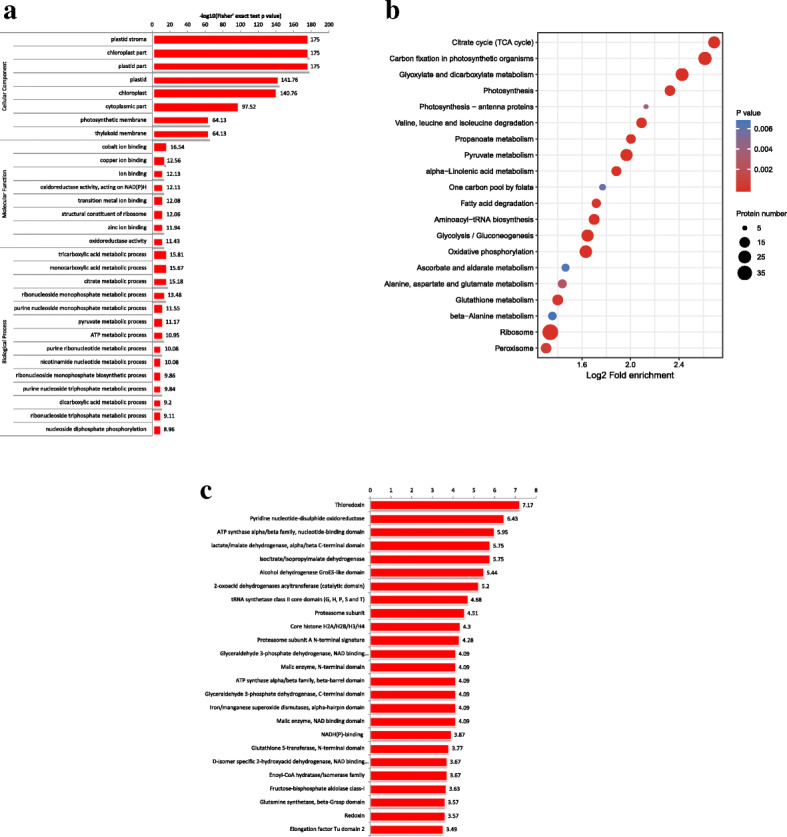
GO-based enrichment analysis in terms of biological process, molecular function and cell component. **a**. Enrichment based on GO annotation. **b**. Enrichment based on KEGG pathways. **c**. Enrichment based on protein domains

**Fig. 6:**
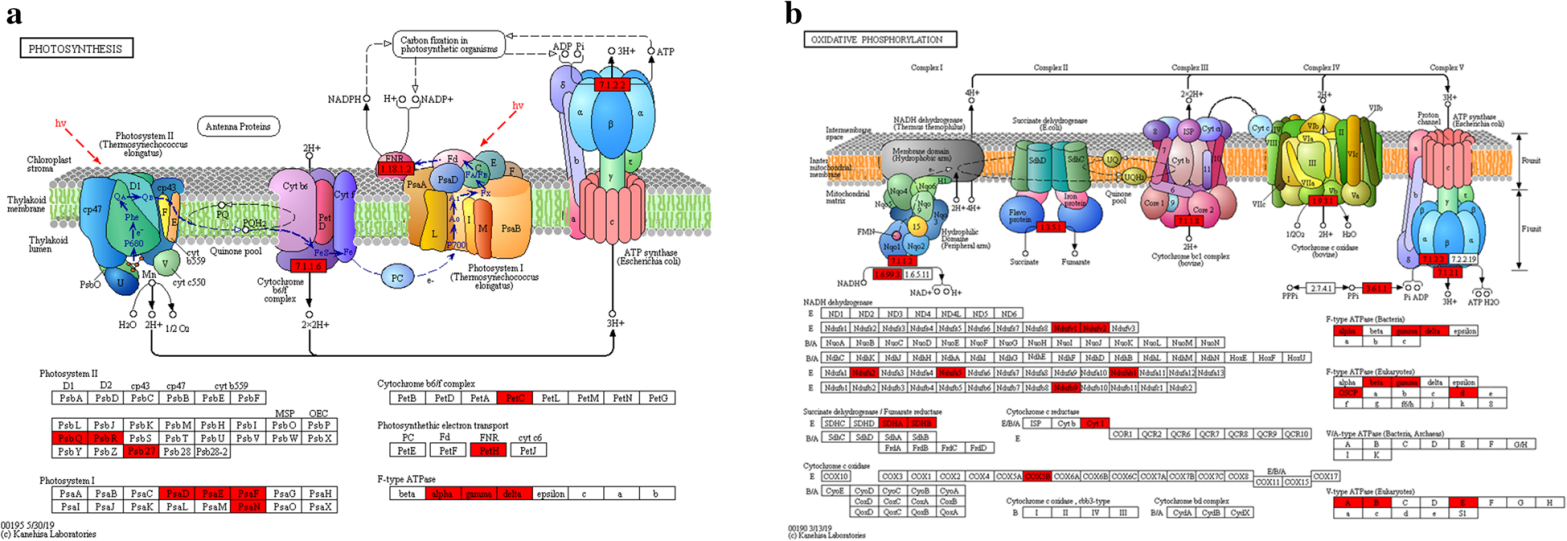
Significantly enriched KEGG pathways in the chloroplast and cytoplasm. **a**. Photosynthesis. **b**. Oxidative phosphorylation. The acetylated proteins are marked in red. The images were generated by KEGG Mapper

The analysis of the subcellular localization of the succinylated proteins showed that most are located in the chloroplast (51.02%), cytoplasm (22.89%), nucleus (9.2%), mitochondria (8.66%) and other compartments (5.24%). Previous studies have demonstrated that lysine succinylation is widespread in chloroplasts [31, 50]. Considering the enrichment of succinylated proteins in energy metabolism (Fig. 5), lysine succinylation may play an important role in photosynthesis. As shown in Fig. 6a, almost all major complexes of the photosynthetic electron transport systems participate in light reactions, including photosystem II, cytochrome b6/f complex, carbon fixation in photosynthetic organisms, photosystem I and ATP synthase; these proteins are succinylated at some subunits or components, such as chlorophyll a/b binding protein and oxygen-evolving enhancer protein 3–2.

We identified 14 succinylated proteins involved in oxidative phosphorylation in the chloroplast (Fig. 6b), including ATPase, cytochrome oxidase and reductase and NADH dehydrogenase. Oxidative phosphorylation plays a central role in various stress responses of many plant species by altering gene expression or protein activity. Lysine succinylation sites in complex I (NADH dehydrogenase), complex II (succinate dehydrogenase), complex III (cytochrome bc1), complex IV (cytochrome c oxidase) and complex V (ATP synthase) may regulate protein interactions between these subunits and influence the production of ATP.

The citrate cycle (TCA cycle or Krebs cycle) is an important aerobic pathway for the final steps of oxidation of carbohydrates and fatty acids. The tricarboxylic acid cycle is a cycle of enzymatic reactions, and the corresponding enzymes are essential for optimal functioning of primary carbon metabolism in plants [51]. TCA cycle pathway enzymes, including succinate-CoA ligase [ADP-forming] subunit (SCOA), succinate dehydrogenase [ubiquinone] flavoprotein subunit (SDH1), succinate dehydrogenase [ubiquinone] iron-sulfur subunit 2 (SDH2–2), dihydrolipoamide acetyltransferase (LAT2 and LTA3), aconitate hydratase 3 (ACO3), leghemoglobin reductase (FLBR), pyruvate dehydrogenase E1 component subunit (PHD2), 3-isopropylmalate dehydrogenase (IDH1), violaxanthin de-epoxidase (VDE1) and 2-oxoglutarate dehydrogenase (ogdh), are succinylated during the growth of paper mulberry (Additional file 4: Table S4 and Fig. 5). The results of the KEGG pathway enrichment showed that 17 protein components or the subunits of the proteins involved in the citrate cycle or enzyme complexes are modified by succinylation; the modifications were characterized by changes in the number of succinylation sites in the conformation trees in paper mulberry, suggesting that lysine succinylation may be involved in the regulation of the citrate cycle in this woody plant.

### Protein–protein interaction network of succinylated proteins in paper mulberry

The identification of protein-protein interaction networks by bioinformatics analysis is a useful tool for formulating testable hypotheses about the functions of uncharacterized proteins. A protein-protein interaction (PPI) network is a network of bimolecular relationships that play important roles in biological activities. Therefore, the analysis of protein interactions and interaction networks is important for understanding the organization, processes and functions of cells [52].

**Fig. 7:**
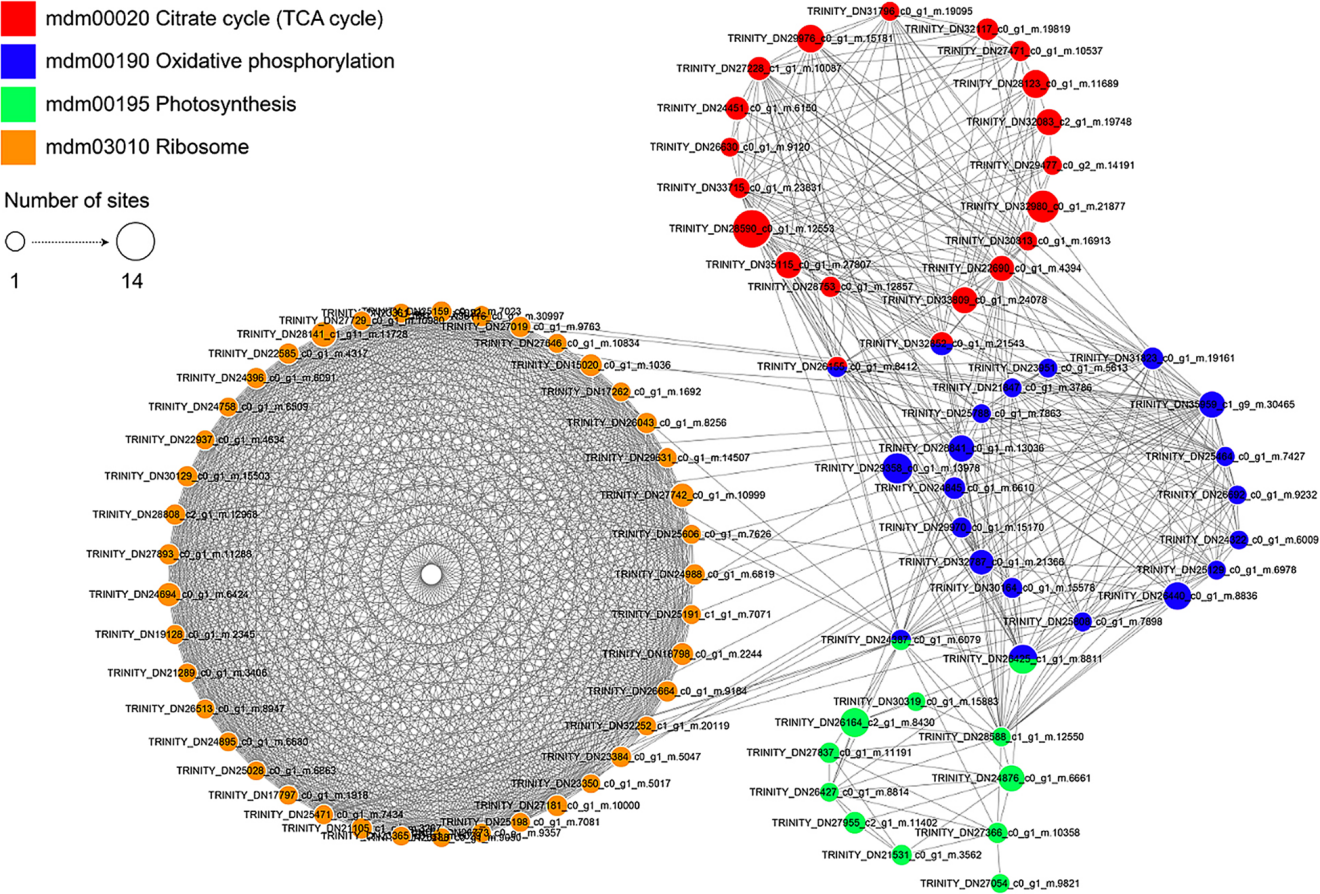
Interaction networks of the succinylated proteins in paper mulberry

To investigate the interactions of various succinylated proteins and their involvement in various interaction pathways, a PPI network of all succinylated proteins was generated that included a total of 90 succinylation sites related to interactions in the protein network (Fig. 7, Additional file 5: Table S5). By defining an algorithm for highly enriched interaction clusters, four highly interconnected succinylated protein clusters were identified. Proteins containing succinylation sites were aggregated into four highly interconnected networks, including the TCA cycle, oxidative phosphorylation, photosynthesis and ribosomes. The results of the protein interaction network analysis indicated that succinylation plays a key role in the regulation of biological processes. Interactions between succinylated proteins and network interactions are complex. Some succinylated proteins are located at the nodes of the interaction network, indicating that the four biological processes are crosslinked and that succinylated proteins coordinate these crosslinks.

A protein interaction network simplifies various complex systems into a set of nodes and edges connecting the nodes. The node degree is the key parameter used to assess the importance and correlation of proteins in a network. Four protein nodes in the network had degrees ≥40 (Additional file 4: Table S5). The results of the protein interaction network analysis showed that lysine succinylation is involved in four processes, namely the TCA cycle, oxidative phosphorylation, photosynthesis and ribosomes, and influences active protein interactions that may play a regulatory role in biological processes in paper mulberry.

## Conclusion

This study identified 2097 succinylation sites in 935 proteins and expanded the scope of lysine succinylation in plants. Motif analysis of succinylated peptides identified four conserved sequence motifs. Analysis of the subcellular localization of succinylated proteins indicated that most succinylated proteins in paper mulberry are distributed in the chloroplast and cytoplasm. Functional analysis revealed that succinylated proteins are involved in diverse metabolic pathways, such as photosynthesis, oxidative phosphorylation and other cellular processes. Photosynthetic processes, including light reactions and carbon fixation, may be regulated by lysine succinylation. Our study confirms the concept that lysine succinylation plays a key regulatory role in various aspects of plant cell metabolism, particularly in photosynthesis and the Calvin-Benson cycle. The dataset can serve as a resource for studies of the function of lysine succinylation in this important woody plant.

## Supplementary Information

**Additional file 1: Table S1.** The identified succinylated sites in paper mulberry leaves.

**Additional file 2: Table S2.** ident Classification in this study.

**Additional file 3: Table S3.** KEGG_pathway_enrichment.

**Additional file 4: Table S4.** Identified succinylated proteins involved in the TCA cycle pathway.

**Additional file 5: Table S5.** The detailed information of TCA cycle, oxidative phosphorylation, photosynthesis, ribosome succinylated proteins involved in Interaction network.

## Data Availability

The data sets supporting the results of this article are included within the article and Additional files.

## Abbreviations

PTMs: Post-translational modifications
LC-MS/MS: Liquid chromatography-mass spectrometry
UPLC: Ultra Performance Liquid Chromatography
GO: Gene ontology
KEGG: Kyoto encyclopedia of genes and genomes
TCA cycle: Citrate cycle
